# Study of fast and slow consecutive processes by heterogeneous isotope exchange using P-32 radiotracer

**DOI:** 10.1007/s10967-018-6231-4

**Published:** 2018-10-05

**Authors:** Noémi M. Nagy, József Kónya

**Affiliations:** Imre Lajos Isotope Laboratory, Department of Physical Chemistry, University of Debrecen, Egyetem tér 1, Debrecen, 4010 Hungary

**Keywords:** Adsorption, Heterogeneous isotope exchange, Kinetics of phosphate sorption, Soil, ^32^P tracer

## Abstract

The sorption of phosphate on soils is studied by radioisotopic tracer method. Two consecutive processes with rather different rates were differentiated: namely the heterogeneous isotope exchange between the phosphate in the soil solution and the weakly sorbed phosphate (fast reaction), and the transformation of weakly sorbed phosphate to tightly sorbed phosphate (slow reaction). In this paper, it is shown how the rate constants of these two processes can be determined by a radiotracer with a relatively short half-life.

## Introduction

Interactions in solid/liquid systems are determined by chemical environment and physical, physicochemical, chemical processes. In some systems, processes take place in different time scales, resulting in different species both in solid and liquid. This may be the case for such a long-time process as the nutrient cycle of soils, mainly determined by the concentration and speciation of macro and micro nutrients in the soil solution as well as the interactions between the solid phase of soil and the soil solution. These interactions are essential in case of nutrients, such as phosphate anion, strongly interacting to some soil components. Phosphate ions can be sorbed on soil by different mechanisms, namely weakly, tightly sorbed and precipitated phosphate species are mentioned [1–5]. The quantity of phosphate uptaken by plants as well as the optimal fertilization depends on the ratio of these phosphate species. The dissolved in soil solution and weakly sorbed phosphate is considered as utilized by plants. The ratio of tightly sorbed phosphate can be significant, but in slow processes this tightly sorbed phosphate can transform to weakly sorbed and dissolved species and vice versa. Besides plantation, optimal fertilization is important because of the limited quantity of raw phosphate which, according to recent estimates, is sufficient for about 70 years.

The ratio of water soluble/weakly sorbed phosphate can be studied by heterogeneous isotope exchange using P-32 labeled phosphate ions. Isotope exchange experiments enable the study of heterogeneous systems under a steady-state. The well-known models of the kinetics of isotope exchange consider the steps of heterogeneous reactions such as diffusion (in solution and solid), chemical exchange, or building into the crystalline lattice, or the combination of these steps as rate determining process [6]. The exchange studies give useful information because there is no chemical change, that is the free enthalpy of the process is zero, the process is directed only by the mixing entropy of the distribution of a radioactive isotope between the phases.

The quantity of water soluble/weakly sorbed phosphate as well as the fast transport rate of phosphate in a steady state between the soil and soil solution can be determined by heterogeneous isotope exchange. In our previous work [5], a review of the studies on phosphate sorption of soils, including heterogeneous isotope exchange studies has been given and critically evaluated. In addition, a correct thermodynamic and mathematical model for the evaluation of the heterogeneous isotope exchange of the radioactive P-32 isotope have been constructed and tested. During the improvement of our model, the previous models describing heterogeneous isotope exchange has been impleted.

In this work, we will illustrate how the fast (minutes) and slow processes (days, weeks) of phosphate sorption can simultaneously be studied by a radioactive tracer method if we have only a tracer with relatively short half-live such as P-32 (*t*_1/2_= 14.26 days). The fast process is the heterogeneous isotope exchange between the phosphate dissolved in the soil solution and weakly sorbed phosphate under a steady state. The slow process is the transformation of weakly to tightly sorbed phosphate.

## Experimental

Two soil samples, a humous sand (calcareous) and a calcareous chernozem was collected in Őrbottyán and Debrecen-Látókép, respectively, Hungary. The total phosphorous content of the soils was 700 and 925 mg/kg, respectively. The details of the experiments have been published elsewhere [5], here only a short description is given. The soil samples were incubated non-radioactively and radioactively by adding different quantities of phosphate (0, 40, 80, 160, and 320 µg P/g soil) as KH_2_PO_4_ solution. During the radioactive incubation, carrier-free ^32^P (as H_3_PO_4_) was added to KH_2_PO_4._ The time of incubation of the radioactive soil samples was limited by the half-life of ^32^P, the incubation time was from 1 to 13 weeks. (13 week incubation resulted in the decrease of the original P-32 activity to about 1 per cent, thus the added activity had to be 100 times higher than the desired activities after incubation. The background activity is about 20 cpm, so a sample had to be at least 200 cpm.)

The incubation times of non-radioactive soils were up to 22 weeks. After incubations, batch studies were done. The non-radioactively incubated soil samples (1 g) was equilibrated with water (200 cm^3^) to reach a steady state (120 min mixing), than carrier-free ^32^P (as H_3_PO_4_) solution was added to the non-radioactive samples; that is the heterogeneous isotope exchange of phosphate between the dissolved and weakly sorbed phosphate was studied. The dissolution of phosphate, including weakly and tightly sorbed species, of the radioactively incubated soils was also studied by batch technique (1 g soil and 200 cm^3^ solution). The phosphate concentration of the solution was determined by the ammonium phospho molybdate spectrophotometric method. The radioactivity of solution was determined by liquid scintillation.

## Results and discussion

**Fig. 1:**
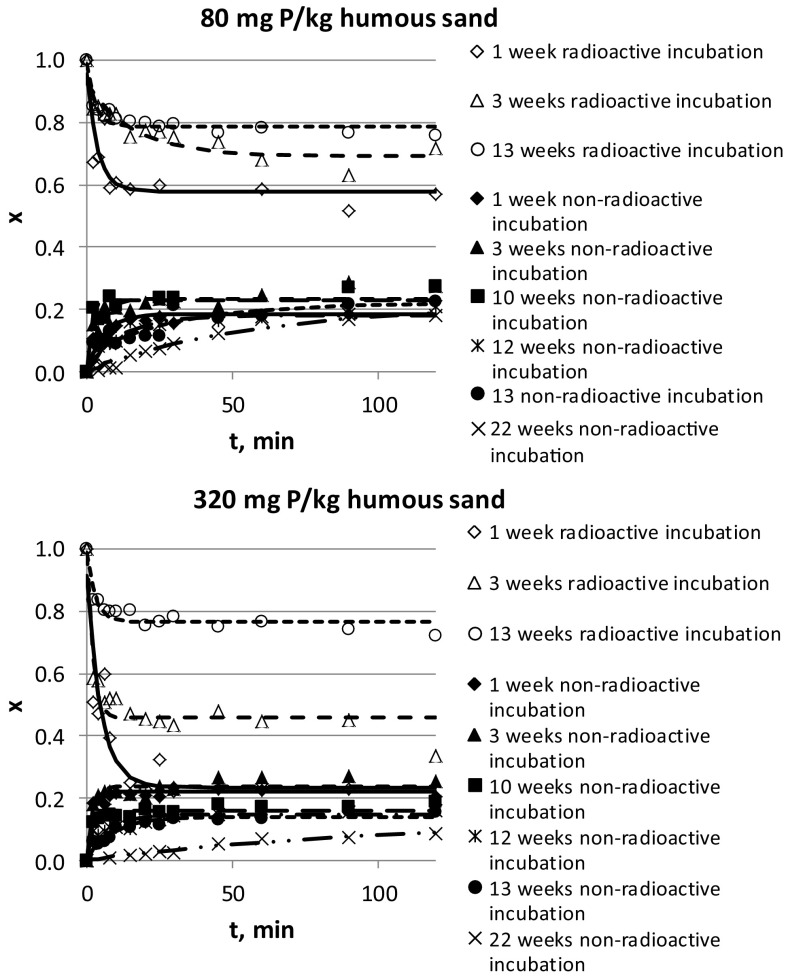
Relative radioactivities of humous sand (calcareous) soil (*x*) non-radioactively and radioactively incubated for 1, 3, 10, 12, 13, and 22 weeks with 80 and 320 μg P/g soil as a function of time. The open symbols are the results of dissolution of phosphate from radioactively incubated soils. The full symbols show the kinetics of the heterogeneous isotope exchange when the carrier-free radioactive phosphate is added to the non-radioactively incubated soils from the solution. The experimental errors of *x* values are ± 3%

Both soil types showed similar tendencies at all added phosphorous quantities (0–320 µg P/g soil), thus only two examples of ten sets of experiments are shown in Fig. 1.

In Fig. 1, the relative radioactivities of the soils (*x *= radioactivity of soil/total radioactivity of the system) are shown as a function of time. Since all experimental conditions were the same during the incubation and batch studies, except that carrier-free phosphate was added to the soil (radioactive incubations) or to the solution (non-radioactive incubations), the plots for non-radioactive and radioactive incubations should coincide when the heterogeneous isotope exchange and dissolution reach the equilibrium state. As shown, however, that the relative radioactivities of soils are usually different, depending on whether the soils were incubated radioactively or non-radioactively, that is depending on whether the carrier-free ^32^P was introduced into the system by soil (radioactive incubation) or the solution (non-radioactive incubation). In addition, as the incubation time increases, the curves of non-radioactive and radioactive incubations get farther away. This difference in the distribution of ^32^P comes from the fact that the two ways of incubation provide different information. During the non-radioactive incubation, all sorption processes of phosphate can occur, both the weakly and tightly sorbed species are present. After the incubation, during the heterogeneous isotope exchange studies, the radioactive ^32^P exchanges only with the weakly sorbed phosphate:

1 $${\text{S}} - {\text{PO}}_{4} {\text{H}}^{ - } + {\text{H}}_{2} {}_{{}}^{32} {\text{PO}}_{4}^{ - } \rightleftarrows {\text{S}} - {}_{{}}^{32} {\text{PO}}_{4} {\text{H}}^{ - } + {\text{H}}_{2} {\text{PO}}_{4}^{ - }$$

where *S* means the surface of soil. The relative radioactivity of soil in this case (*x*_HIE_) is expressed as:

2 $$x_{\text{HIE}} = \frac{{m_{2} }}{{m_{1} + m_{2} }}$$

where *m*_*1*_ and *m*_*2*_ are the phosphorous quantities of the soil solution and weakly sorbed phosphorous in soil, respectively.

The quantity of the tightly sorbed phosphorous (*m*_tightly_) is obtained as.

3 $$m_{\text{tightly}} = P_{\text{total}} - (m_{1} + m_{2} )$$

where *P*_total,_ is the total phosphorous quantity of soil (the phosphorous content of the original soil + the added phosphorous for incubation).

During the radioactive incubation, the ^32^P tracer takes place in all sorption processes, it is present as weakly and tightly sorbed species, and during the dissolution studies the relative radioactivity of the soil (*x*_*D*_) is as follows:

4 $$x_{D} = \frac{{m_{2} + m_{\text{tightly}} }}{{m_{1} + m_{2} + m_{\text{tightly}} }} = \frac{{m_{2} + m_{\text{tightly}} }}{{P_{\text{total}} }}$$

As seen from Eqs. (2) and (4), if the quantity of the tightly sorbed phosphate (*m*_tightly_) were zero (no transformation of weakly to tightly sorbed phosphate), *x*_HIE_= *x*_*D*_. Since it is not the case (Fig. 1), it is obvious that a part of the phosphate during the incubation transforms to tightly sorbed species and the ratio of the tightly sorbed phosphate species increases as the incubation time increases.

Figure 1 shows too that the relative radioactivities of the radioactively incubated soils become more and more as the incubation time increases, proving the weakly to tightly transformation of sorbed phosphate species. The relative radioactivities of the non-radioactively incubated soils, however, change in a less degree because in this case only the weakly sorbed and the dissolved phosphate takes place in the heterogeneous isotope exchange.

The equilibrium relative radioactivities of the soils incubated by the two ways are the closest at 320 µg P/g soil (both for humous sand and chernozem) and 1 week incubation because the weakly to tightly transformation is a slow process and the added phosphate quantity is relatively high compared to the tightly sorbed phosphate of the original soil. At 160 µg P/g chernozem, similar result is obtained (i.e., the equilibrium relative radioactivities of chernozem soil are very close), but in case of 160 µg P/g humous sand, the curves are very far from each other, showing that the weakly to tightly transformation is faster in case of humous sand than in case of chernozem.

On the basis of these results, the scheme of the phosphate sorption and the interactions between the soil and solution is expressed as:

5 $$P_{\text{soil solution}} \begin{array}{*{20}c} {\text{short time}} \\ \rightleftarrows \\ {\text{heterogeneous isotope exchange, dissolution}} \\ \end{array} P_{\text{soil weakly sorbed }} \begin{array}{*{20}c} {\text{long time}} \\ \rightleftarrows \\ {\text{incubation}} \\ \end{array} P_{\text{tightly}}$$

The *x*-*t* curves (e.g., Fig. 1) can quantitatively be evaluated. For the dissolution of phosphate after radioactive incubations, simple first-order kinetics is applied.

For the heterogeneous isotope exchange [5] after non-radioactive incubations the kinetic equation can be derived:

6 $$x_{\text{HIE}} = \frac{{m_{2} }}{{m_{1} + m_{2} }}\left( {1 - \exp \left( { - \frac{C}{{m_{1} }}\frac{{m_{1} + m_{2} }}{{m_{2} }}t} \right)} \right)$$

Equation (6) expresses that *m*_*2*_/(*m*_*1*_+ *m*_*2*_), *C,* the rate as well as *C*/*m*_*1*_, the rate constant of the heterogeneous isotope exchange (expressed in µg min^−1^ or min^−1^, respectively) can be calculated from the relative radioactivities of the soil (*x*_HIE_) versus time plots [5].

Equation (6) is a first-order kinetic equation, thus the half-life (min) of the short time process, namely the heterogeneous isotope exchange between the soil solution and weakly sorbed phosphate, can be calculated by the usual formula for first-order kinetics:

7 $$t_{1/2}=\frac{{\text{ln}}\,2}{C/m_1}$$

The experimental results show that the rate constants of heterogeneous isotope exchange are in the range of 8 × 10^−2^–3 × 10^−2^ min^−1^. This means that the half-life of the heterogeneous isotope exchange is in the range of 8–20 min.

When the phosphorous quantity in the solution (*m*_*1*_ (µg)) is measured by an independent analytical method (e.g., spectrophotometry), the exchange rate of phosphate between the soil solution and weakly sorbed species in steady state (*C,* µg/min) and the quantity of weakly sorbed or water soluble/exchangeable phosphorous in the soil (*m*_*2*_, (µg) can be calculated.

**Fig. 2:**
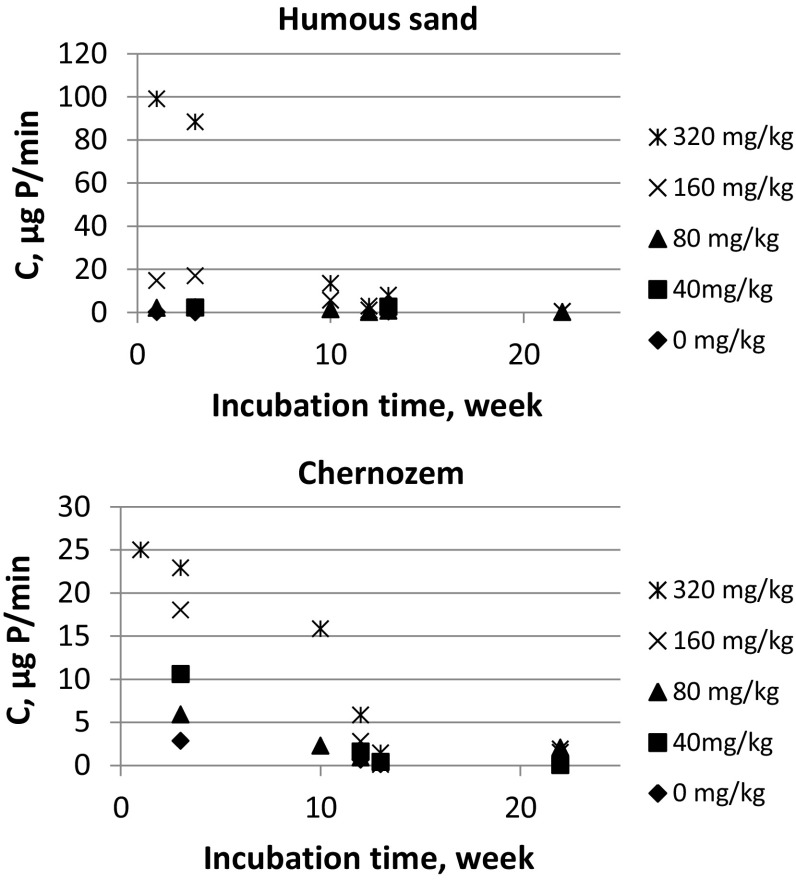
The exchange rates of heterogeneous isotope exchange between the dissolved and weakly sorbed phosphate on humous sand and chernozem at different added phosphate quantities as a function of incubation time. The experimental errors of *C* values are ± 10%

This means that the *C,* the rate of the short time process can directly be calculated (Eq 6). The *C* values of for the studied soils at all phosphorous quantities and incubation times are summarized in Fig. 2. The interval of the rates is typically in the range of some µg P/min to some 10 µg P/min.

As seen in Fig. 2, the exchange rate of phosphate between the soil solution and the weakly sorbed phosphate decreases as the incubation time increases, proving the transformation of phosphate from the weakly to tightly sorbed species during the incubation. At each incubation time, the rate of exchange increases as the quantity of the added phosphate increases.

**Fig. 3:**
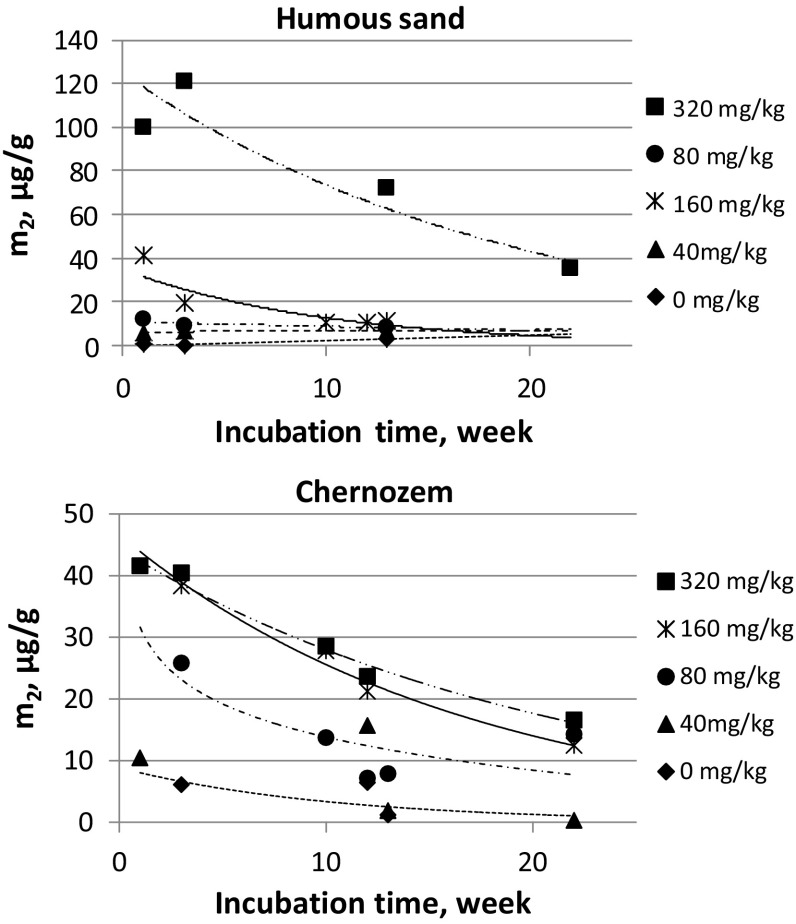
Weakly sorbed phosphorous quantity in soil (*m*_*2*_) for humous sand and chernozem at different phosphate quantities as a function of incubation time. The experimental errors of *m*_*2*_ values are ± 10%

The *m*_*2*_ values for the studied soils at all phosphorous quantities and incubation times are illustrated in Fig. 3.

As seen in Fig. 3, the quantity of the weakly sorbed phosphate *m*_*2*_ increases as the quantity of added phosphate increases. Moreover, the *m*_*2*_ decreases as the incubation time increases.

The *m*_2_-*t* functions (Fig. 3) make possible to calculate the rate of the weakly to tightly sorbed phosphate transformation. Based on the well-known formula of consecutive reactions [7], the rate of the long time (slow) reaction can be calculated by a simple first-order kinetics, if the rate of the short time (fast) reaction is much less than the rate of the long time (slow) reaction. Equation (5) shows that this requirement is fulfilled in our system. We have a consecutive reaction where the half-life of the short time reaction, the heterogeneous isotope exchange, is in the range of minutes. The time interval of the long time reaction, the transformation of weakly to tightly sorbed phosphate can be expressed in weeks as seen in Fig. 3. Quantitatively, in case of chernozem and humous sand, at low phosphorous quantities (0 and 40 µg P/g soil), the half-life of the weakly to tightly sorbed phosphate transformation is about 7 weeks (rate constant about 0.1 week^−1^) and shorter than 1 week (rate constant is longer than 7 × 10^−1^ week^−1^), respectively. At high phosphorous quantities (80, 160, and 320 µg P/g soil), the half-lives are longer than 10 weeks (rate constant is shorter than 7 × 10^−2^ week^−1^), or in the range of 4–9 weeks (rate constant is shorter than 1.8 × 10^−2^–7.6 × 10^−2^ week^−1^), for chernozem and humous sand, respectively.

We would like to compare our rate constants or half-lives with previous literature data, but we did not find such values. Phosphate exchange of soils, however, has been studied [8, 9], but the kinetics of heterogeneous isotope exchange was not characterized by usual rate constant but an empirical formula using the exchanged ratios at 1 min and in equilibrium. The formula includes a parameter (*n*) characteristic to the kinetics of the process. In a previous paper [5], we criticized this treatment, but now we tried to apply the formula to our results. The experiences showed that when the exchanged ratio at 1 min and in equilibrium as well as the n parameter are treated as parameters, the value of the exchanged ratio at 1 min is very far from the real experimental data (e.g., 0.3 is obtained, while the experimental data is 0.95). Thus, the parameter fitting is uncertain.

## Conclusions

A consecutive process consisting of two steps with rather different rates can be studied using one radioactive isotope with a relatively short half-life. At a steady state, fast reaction (first step) is studied by heterogeneous isotope exchange. The distribution of the reactants at a steady state as well as the rate constant of the steady state reaction can directly be calculated from the kinetics of the heterogeneous isotope exchange; that is from the change of the relative radioactivity as a function of relatively short time. The second slow step is studied so that this process is permitted to occur without radioactive isotope (incubation). Then, heterogeneous isotope exchange studies are done after any long incubation time (not limited by the decay of the tracer), determining the steady-state distributions and rate constants at these different incubation times. The rate of the slow process can be calculated from the change of the distribution of the reactants as a function of incubation time.

The incubation can be done radioactively, too, but in this case the incubation time is limited by the decay of the radioactive isotope. However, the comparison of the results of the radioactive and non-radioactive incubations in this limited time interval also shows the existence of the slow process.

In this paper, the sorption of phosphate on two types of soil (humous sand and chernozem) was shown as an example. However, the method can be applied in other systems where two consecutive processes with rather different rates take place.

The result of these studies may be useful in field application of phosphate fertilizers, that is the addition of small quantities of phosphate at multiple time points may be preferable other that adding the lot.
